# Spectral Data Analysis and Identification of Vancomycin Hydrochloride

**DOI:** 10.3389/fchem.2021.753060

**Published:** 2021-09-20

**Authors:** Ye Tian, Xiaomeng Chong, Shangchen Yao, Mingzhe Xu

**Affiliations:** National Institutes for Food and Drug Control, Beijing, China

**Keywords:** NMR, MS, chemical shift, vancomycin, structure elucidation

## Abstract

**Objective:** To establish a method for the determination of the chemical structure of vancomycin hydrochloride.

**Methods:** Nuclear magnetic resonance spectroscopy and mass spectrometry were conducted to analyze the chemical structure of vancomycin hydrochloride.

**Results:** In this study, the target compound (1) was identified as (Sα)-(3S, 6R, 7R, 22R, 23S, 26S, 36R, 38αR)-44-[[2-O-(3-amino-2, 3, 6-trideoxy-3-C-methyl-α-L-lyso-hexopyranosyl)-β-D-glucopyranosyl] oxy]-3-(carbamoylmethyl)-10, 19-dichloro-7, 22, 28, 30, 32-pentahydroxy-6-[[(2R)-4-methyl-2-(methylamino) pentanoyl] amino]-2, 5, 24, 38, 39-pentaoxo-2, 3, 4, 5, 6, 7, 23, 24, 25, 26, 36, 37, 38, 38α-tetradecahydro-22H-8, 11: 18, 21-dietheno-23, 36-(iminomethano)-13, 16: 31, 35-dimetheno-1H, 13H-[1, 6, 9] oxadiazacyclohexadecino [4, 5-m] [10, 2, 16]-benzoxadiazacyclotetracosine-26-carboxylic acid hydrochloride.

**Conclusion:** The method used in this study is accurate and can be used for the production and structural elucidation of vancomycin hydrochloride.

## Introduction

**FIGURE 1:**
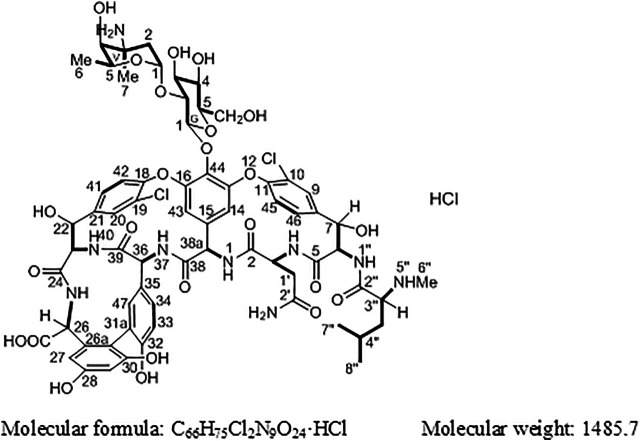
Structure of vancomycin hydrochloride (1).

Vancomycin hydrochloride (Figure 1) is a glycopeptide antibiotic that exhibits significant antibacterial activity against various Gram-positive bacteria, including cocci and bacilli (Mccormick et al., 1956). The mechanism of action of vancomycin hydrochloride is as follows: vancomycin binds to the alanine moiety of the precursor peptide present on the sensitive bacterial cell wall with high affinity, thereby preventing the synthesis of the polymer peptidoglycan that constitutes the bacterial cell wall, resulting in cell wall defects and bacterial death. Existing literature shows that vancomycin can selectively inhibit the synthesis of ribonucleic acid by altering the permeability of bacterial cell membranes (Wang et al., 2018). This property is listed in the Chinese Pharmacopoeia (2020 edition, Volume II), Japanese Pharmacopoeia (17th edition), United States Pharmacopoeia (43rd edition), and European Pharmacopoeia (10.0 edition).

China is an important supplier of the raw materials used in vancomycin synthesis. Presently, to the best of our knowledge, the structural analysis of vancomycin has not been reported. Moreover, due to its complex structure, the signals obtained in the nuclear magnetic resonance (NMR) spectrum of vancomycin tend to overlap, making it difficult to analyze and elucidate its structure. In this study, mass spectrometry (MS), one- (^1^H, ^13^C) and two-dimensional (^1^H-^1^H) correlation spectroscopy (COSY), and heteronuclear multiple quantum coherence (HMQC) and heteronuclear multiple bond coherence (HMBC)) NMR spectroscopy were comprehensively performed to analyze the structure on the basis of the spectral data of vancomycin hydrochloride. The obtained results will provide a scientific basis for the qualitative analysis of this compound.

## Materials and Methods

### Reagents

Formic acid (Fluka, GR), acetonitrile (Fisher, HPLC), Watsons purified deionized water (Xellia pharmaceuticals, batch number: A3250080), and vancomycin hydrochloride reference standard (National Institutes for Food and Drug Control, batch number: 130360-201302) were used in this study. Deuterated dimethyl sulfoxide (Merck) was used for NMR analysis of 1.

### Instruments

Mass spectra were recorded using an AB Sciex 3200 Qtrap mass spectrometer (AB SCIEX, United States). NMR spectra were obtained using a Varian INOVA 600 MHz NMR spectrometer.

## Results

Compound 1 is a white solid. The positive ion electrospray ionization mass spectra [(+)-ESIMS] of 1 revealed a quasi-molecular ion peak at (m/z) 1448 [M+H]+. High-resolution (+)-ESIMS afforded the accurate mass-to-charge ratio of the quasi-molecular ion at m/z 1448.4380 [M+H]+, suggesting that the molecular composition of the test product was C_66_H_75_C_l2_N_9_O_24_ (the calculated value was 1448.4375, and the corresponding molecular composition was C_66_H_76_C_l2_N_9_O_24_). The signals in the ^1^H-NMR spectrum of 1 were assigned to five groups of aromatic proton as follows: 1) three groups of ABX-system aromatic proton: δ_H_ 7.38 (1H, br s, H-9), 7.52 (1H, brd, *J* = 8.4 Hz, H-46), and 7.26 (1H, brd, *J* = 8.4 Hz, H-45); δ_H_ 7.86 (1H, s, H-20), 7.47 (1H, brd, *J* = 8.4 Hz, H-41), and 7.34 (1H, brd, *J* = 8.4 Hz, H-42); δ_H_ 7.18 (1H, br s, H-47), 6.77 (1H, brd, *J* = 8.4 Hz, H-34), and 6.72 (1H, brd, *J* = 8.4 Hz, H-33); and 2) two groups of 3-substituted aromatic proton signals: δ_H_ 6.42 (1H, br s, H-29), 6.27 (1H, br s, H-27), and δ_H_ 5.54 (1H, br s, H-14), 5.21 (1H, br s, H-43)]. Moreover, three signals of methyl protons bonded to methylidyne [δ_H_ 0.90 (3H, d, *J* = 6.6 Hz, H3-8″), 0.86 (3H, d, *J* = 6.6 Hz, H3-9″), and 1.07 (3H, d, *J* = 6.0 Hz, H3-V6)], a signal of methyl protons bonded to a nitrogen atom [δ_H_ 2.37 (3H, s, H3-7″)], a signal of methyl protons bonded to a quaternary carbon [δ_H_ 1.32 (3H, s, H3-V7)], and 19 signals of protons bonded to various heteroatoms [δ_H_ 5.75 (1H, d, *J* = 7.8 Hz, H-38a), 5.27 (1H, d, *J* = 7.2 Hz, H-G1), 5.24 (1H, br s, H-V1), 5.16 (1H, br s, H-7), 5.13 (1H, br s, H-22), 4.88 (1H, br s, H-6), 4.68 (1H, q, *J* = 6.6 Hz, H-V5), 4.43 (1H, o, H-36), 4.42(1H, d, *J* = 6.0 Hz, H-26), 4.35(1H, o H-3), 4.19(1H, d, *J* = 11.4 Hz, H-23), 3.68(1H, d, *J* = 10.2 Hz, H-G6a), 3.57(1H, t, *J* = 8.5 Hz, H-G2), 3.56(1H, o, H-G6b), 3.46(1H, o, H-G3), 3.35(1H, o, H-G5), 3.35 (1H, o, H-G4), 3.35 (1H, o, H-3″), and 3.20 (1H, br s, H-V4)] were observed in the NMR spectra of 1. After deuterium exchange, the NMR spectra of 1 exhibited at least 16 exchangeable active proton signals [δ_H_ 9.44 (1H, br s, OH), 9.11 (1H, br s, OH), 8.63 (1H, br s, NH-37), 8.47 (1H, d, *J* = 6.0 Hz, NH-25), 8.25 (1H, br s, NH-1), 7.93 (1H, br s, NH-1″), 7.37 (2H, br s, CONH2), 6.91 (2H, br s, CONH2), 6.67 (1H, d, *J* = 11.4 Hz, NH-40), 6.62 (1H, br s, NH-4), 5.96 (1H, d, *J* = 4.2 Hz, OH-22), 5.83 (1H, br s, OH-7), 5.43 (1H, br s, OH-V4), 5.38 (1H, br s, OH-G3), 5.11 (1H, br s, OH-G4), and 4.05 (1H, br s, OH-G5)]. In addition, multiple aliphatic hydrogen signals were observed in the upfield region. The above spectral data show that 1 is a glycopeptide containing multiple amino acid fragments (Harris et al., 1983; Zhou et al., 2004).

The ^1^H and ^13^C NMR signals of 1 were accurately assigned using HSQC and ^1^H-^1^H COSY; the details are listed in Table 1. The ^1^H-^1^H COSY spectrum of 1 showed the cross-peak correlation between the protons. The arrangement of the structural fragments of 1 was determined on the basis of the proton couplings in the structure (represented by thick lines in Figure 5).

**TABLE 1:** ^13^C-NMR and ^1^H-HMBC data of 1.

No.	Type	Chemical shift (^13^C)	Chemical shift (^1^H)	No.	Type	Chemical shift (^13^C)	Chemical shift (^1^H)
1	NH		8.25 (1H, br s)	36	CH	53.7	4.43 (1H, o)
2	C	170.7		37	NH		8.63 (1H, br s)
3	CH	50.9	4.35 (1H, o)	38	C	169.6	
4	NH		6.62 (1H, br s)	38a	CH	54.9	5.75 (1H, d, 7.8)
5	C	167.2		39	C	169.1	
6	CH	58.3	4.88 (1H, br s)	40	NH		6.67 (1H, d, 11.4)
7	CH	71.1	5.16 (1H, br s)	41	CH	127.3	7.47 (1H, br d, 8.4)
8	C	127.2		42	CH	123.4	7.34 (1H, br d, 8.4)
9	CH	128.7	7.38 (1H, br s)	43	CH	104.6	5.21 (1H, br s)
10	C	139.8		44	C	131.9	
11	C	149.8		45	CH	124.3	7.26 (1H, br d, 8.4)
12				46	CH	127.2	7.52 (1H, br d, 8.4)
13	C	152.2		47	CH	135.7	7.18 (1H, br s)
14	CH	107.0	5.54 (1H, br s)	1′	CH_2_	37.3	2.42 (1H, o); 2.14 (1H, dd, 15.6, 4.8)
15	C	134.5		2′	C	171.0	
16	C	151.3		1″	NH		7.93 (1H, br s)
17				2″	C	173.7	
18	C	148.3		3″	CH	61.8	3.35 (1H, o)
19	C	126.2		4″	CH_2_	40.7	1.53 (1H, m, 6.6); 1.53 (1H, m, 6.6)
20	CH	127.3	7.86 (1H, s)	5″	CH	24.1	1.17 (1H, non, 6.6)
21	C	142.5		6″			
22	CH	71.6	5.13 (1H, br s)	7″	CH_3_	33.2	2.37 (3H, s)
23	CH	61.9	4.19 (1H, d, 11.4)	8″	CH_3_	22.9	0.90 (3H, d, 6.6)
24	C	167.6		9″	CH_3_	22.6	0.86 (3H, d, 6.6)
25	NH		8.47 (1H, d, 6.0)	G1	CH	101.3	5.27 (1H, d, 7.2)
26	CH	56.9	4.42 (1H, d, 6.0)	G2	CH	78.1	3.57 (1H, t, 8.5)
26a	C	136.4		G3	CH	76.7	3.46 (1H, o)
26-COOH	C	172.8		G4	CH	70.1	3.35 (1H, o)
27	CH	105.9	6.27 (1H, br s)	G5	CH	76.7	3.35 (1H, o)
28	C	157.2		G6	CH_2_	61.2	3.68 (1H, d, 10.2); 3.56 (1H, o)
29	CH	102.3	6.42 (1H, br s)	V1	CH	96.8	5.24 (1H, br s)
30	C	156.5		V2	CH_2_	33.2	1.90 (1H, brd, 9.0); 1.75 (1H, brd, 9.0)
31	C	118.0		V3	C	53.9	
31a	C	121.7		V4	CH	70.7	3.20 (1H, br s)
32	C	155.1		V5	CH	63.1	4.68 (1H, q, 6.6)
33	CH	116.2	6.72 (1H, br d, 8.4)	V6	CH_3_	16.8	1.07 (3H, d, 6.0)
34	CH	125.4	6.77 (1H, br d, 8.4)	V7	CH_3_	22.3	1.32 (3H, s)
35	C	126.2					

**FIGURE 2:**
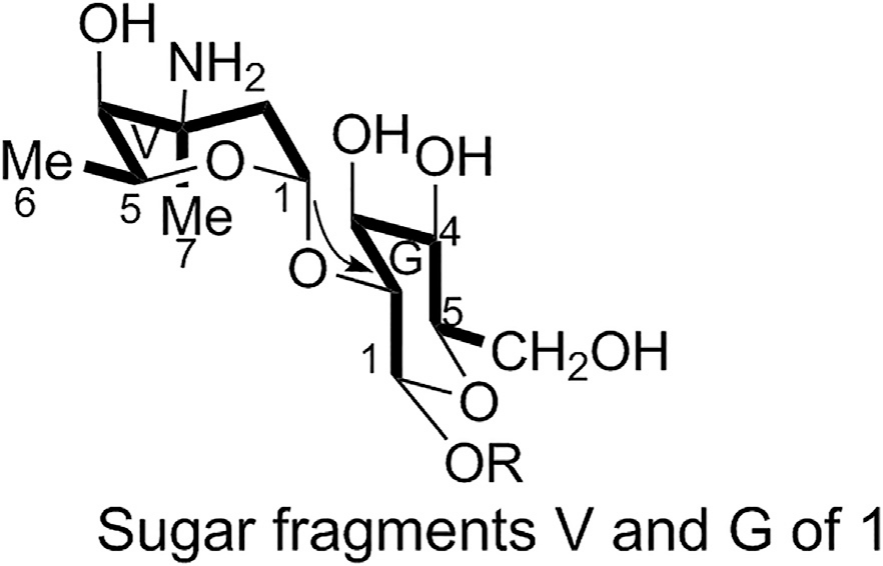
Sugar fragments V and G of 1.

The fragments were then arranged based on the HMBC spectrum as follows:

1) Linkage of sugar fragments:

Two end group carbon signals were observed in the ^1^H-NMR and ^13^C-NMR spectra of 1. The structures and the linkage locations of the two sugar fragments (represented by V and G, respectively in Figure 2) were confirmed based on the HSQC, ^1^H-^1^H COSY, and HMBC spectra. Especially, in the HMBC spectrum, H-V1 can be attributed to C-G2, thereby confirming the α-L-vancosaminyl-(1→2)-O-β-D-glucosyl linkage between the two sugars.

**FIGURE 3:**
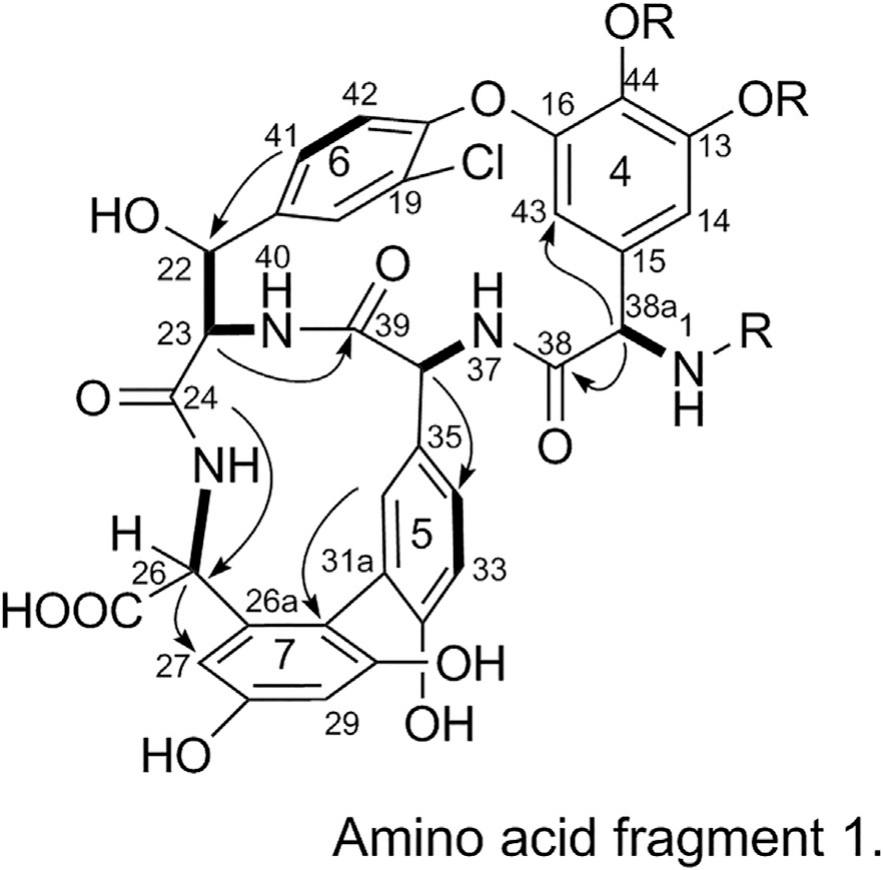
Amino acid fragment 1.

2) Linkage of amino acid fragments 4, 5, 6, and 7:

The substitution patterns of benzene rings 4, 5, 6, and 7 were determined using the δ_H_, δ_C_, and *J*_H-H_ values observed in the ^1^H-NMR and ^13^C-NMR spectra of 1. In the HMBC spectrum of 1, H-38a corresponds to C-43 and C38, H-36 corresponds to C-34, H-41 corresponds to C-22, H-23 corresponds to C-39, and H-26 corresponds to C-24 and C-27. Thus, it can be inferred that 1 contains a 4-amino acid cyclic peptide structure (represented by thick lines in Figure 3).

**FIGURE 4:**
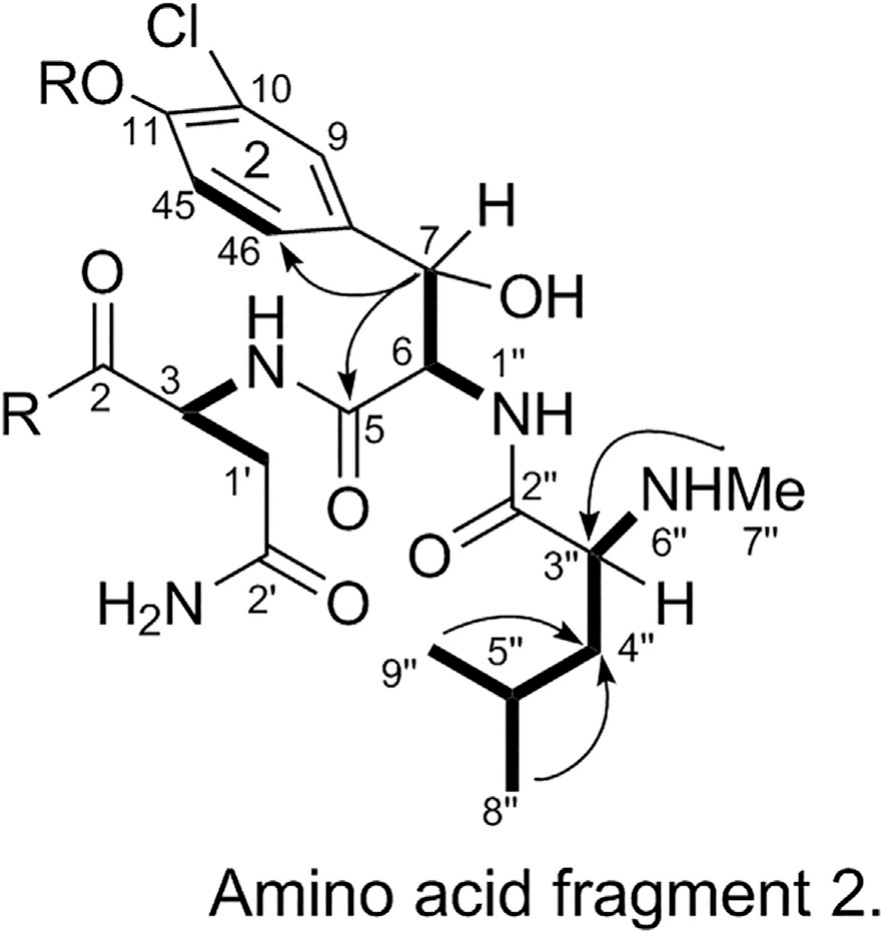
Amino acid fragment 2.

3) Linkage of amino acid fragments 1, 2, and 3:

The substitution pattern of benzene ring 2 was confirmed using the δ_H_, δ_C_, and *J*_H-H_ values observed in the ^1^H-NMR and ^13^C-NMR spectra of 1. In the HMBC spectrum, H_3_-7″ corresponds to C-3″, H-8″ and H-9″ correspond to C-1a, H-7 corresponds to C-11 and C-2, and H-3 corresponds to C-2. Therefore, it could be inferred that there was another 3-amino acid fragment linkage in 1 (represented by thick lines in Figure 4).

4) Linkage of amino acid and sugar fragments.

**FIGURE 5:**
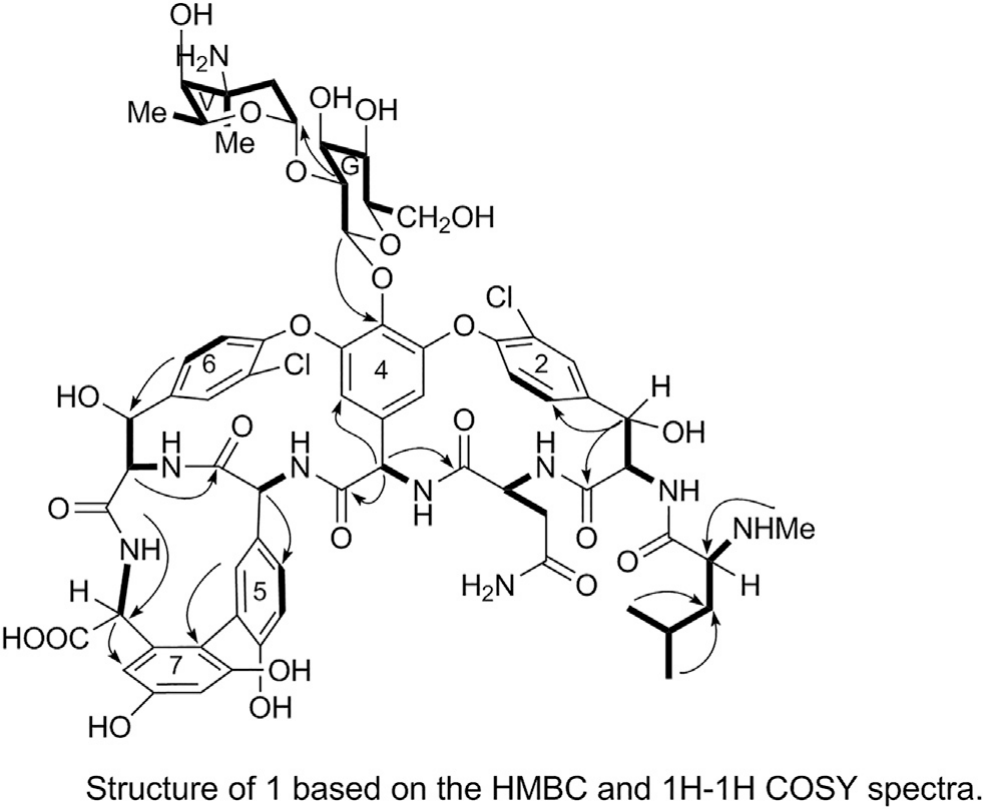
Structure of 1 based on the HMBC and ^1^H-^1^H COSY spectra.

In the HMBC spectrum, H-G1 corresponds to C-44 and H-38a corresponds to C-2. These results confirmed the proposed arrangements of the above-mentioned three fragments (marked by arrows in Figure 5). The above analyses show that 1 is a glycopeptide antibiotic with a cyclic peptide core consisting of seven amino acids and two sugars linked by an oxygen glycosidic bond; its planar structure is identical to that of vancomycin (Mccormick et al., 1956).

**FIGURE 6:**
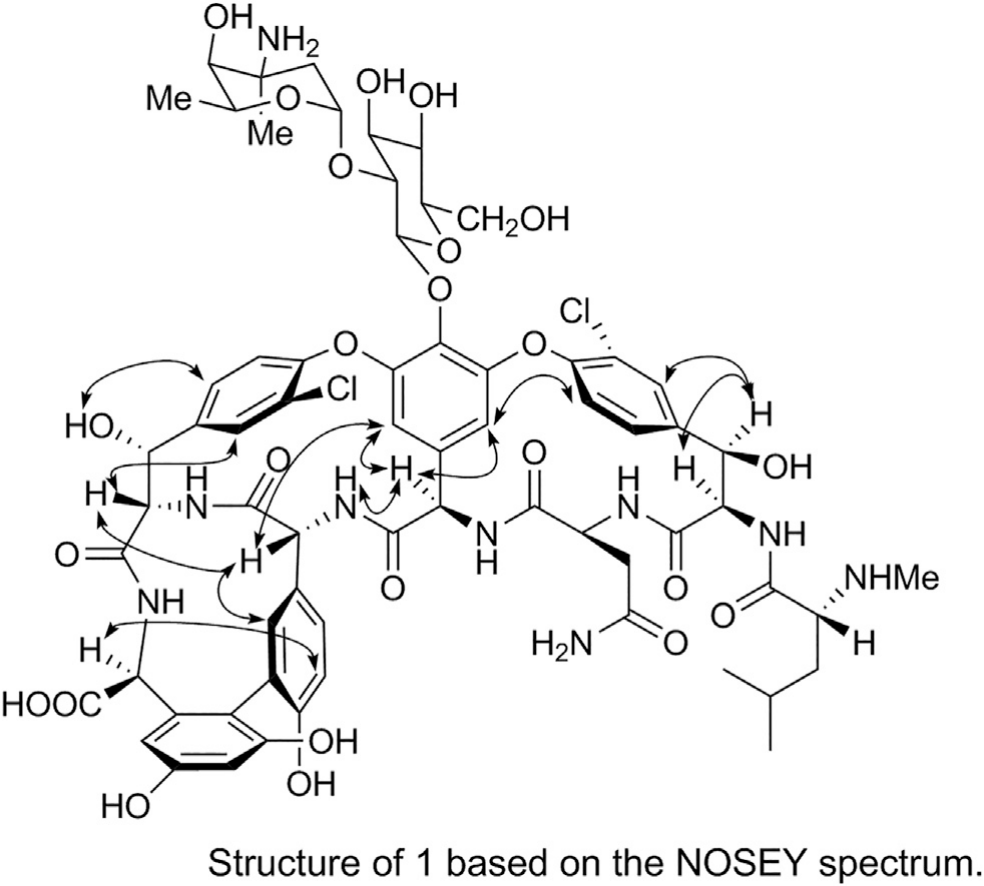
Structure of 1 based on the NOSEY spectrum.

The spatial structure of 1 was determined by comparing its NOSEY and circular dichroism (CD) spectra with those of the reference standard. First, the relative configuration of 1 was determined using the NOSEY experimental data (indicated by arrows in Figure 6). Moreover, the δ_H_, *J*_H-H_, and δ_C_ values and the NOSEY spectra of 1 were consistent with those of the reference standard. These results confirm that 1 has a configuration identical to that of the vancomycin hydrochloride reference standard.

**FIGURE 7:**
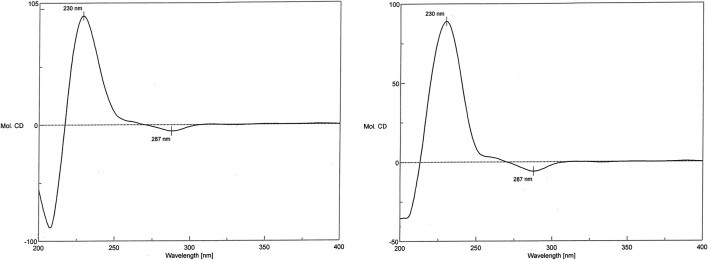
CD spectra of 1 and the reference standard.

In the CD spectrum of 1, a negative Cotton effect was observed at 287 nm, while a positive Cotton effect was observed at 230 nm. The CD spectrum of 1 was identical to that of the vancomycin hydrochloride reference standard (2) (Figure 7). Therefore, 1 exhibited the three-dimensional structure identical to that of the vancomycin hydrochloride reference standard.

## Discussion

Through MS and NMR spectroscopy, 1 was identified as vancomycin hydrochloride. Moreover, the ^1^H and ^13^C NMR signals were accurately assigned on the basis of the one- and two-dimensional NMR spectra of 1. Thus, this method can be effectively utilized for the structural and qualitative analysis of complex compounds.

## Data Availability

The original contributions presented in the study are publicly available. This data can be found in the European Nucleotide Archive (ebi.ac.uk) under accession number PRJEB47449.
