# Supplementary material for: Spectral Data Analysis and Identification of Vancomycin Hydrochloride
Source: Front Chem. 2021 Sep 20;9:753060. doi: 10.3389/fchem.2021.753060 (PMC8488278; doi:10.3389/fchem.2021.753060)
Supplement: Supplementary file 1 [file DataSheet1.PDF]

Spectral data analysis and identification of vancomycin hydrochloride

TIAN Ye[1†], CHONG Xiaomeng[1†], YAO Shangchen[1*], XU Mingzhe[1*]

[1] National Institutes for Food and Drug Control, Beijing 102629, China

Supplementary Content

List of Contents

No.	Content	Page
1	Figure S1. The ^{1}H NMR Spectrum of vancomycin hydrochloride in DMSO- d_6 (600 MHz).	S3
2	Figure S2. The ^{13}C NMR Spectrum of vancomycin hydrochloride in DMSO- d_6 (150 MHz).	S4
3	Figure S3. The ^{1}H-^{1}H gCOSY Spectrum of vancomycin hydrochloride in DMSO-d_6 (600 MHz).	S5
4	Figure S4. The gHSQC Spectrum of vancomycin hydrochloride in DMSO-d_6 (600 MHz).	S6
5	Figure S5. The gHMBC Spectrum of vancomycin hydrochloride in DMSO-d_6 (600 MHz).	S7

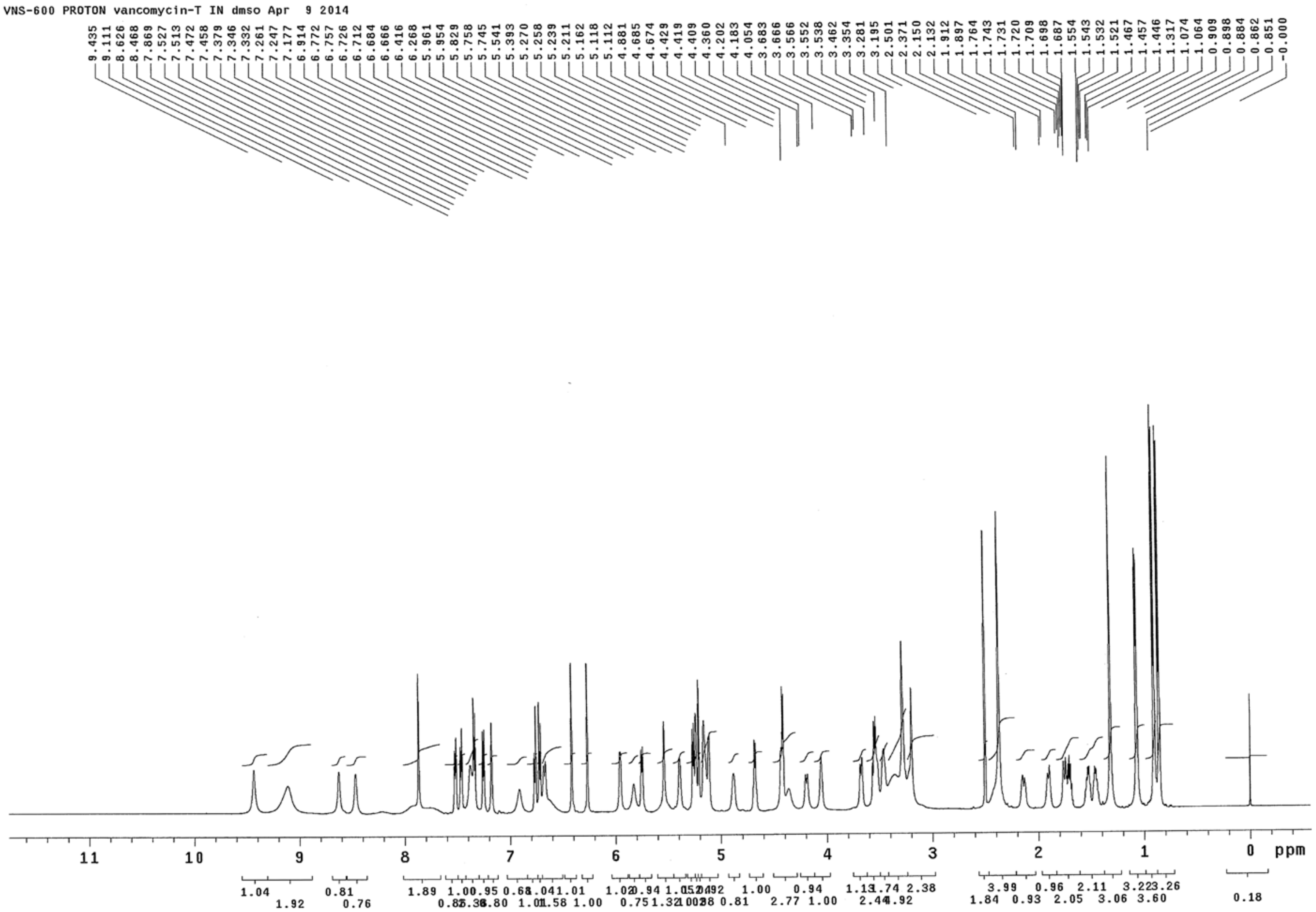

Figure S1. The ^{1}H NMR Spectrum of vancomycin hydrochloride in DMSO-d_6 (600 MHz).

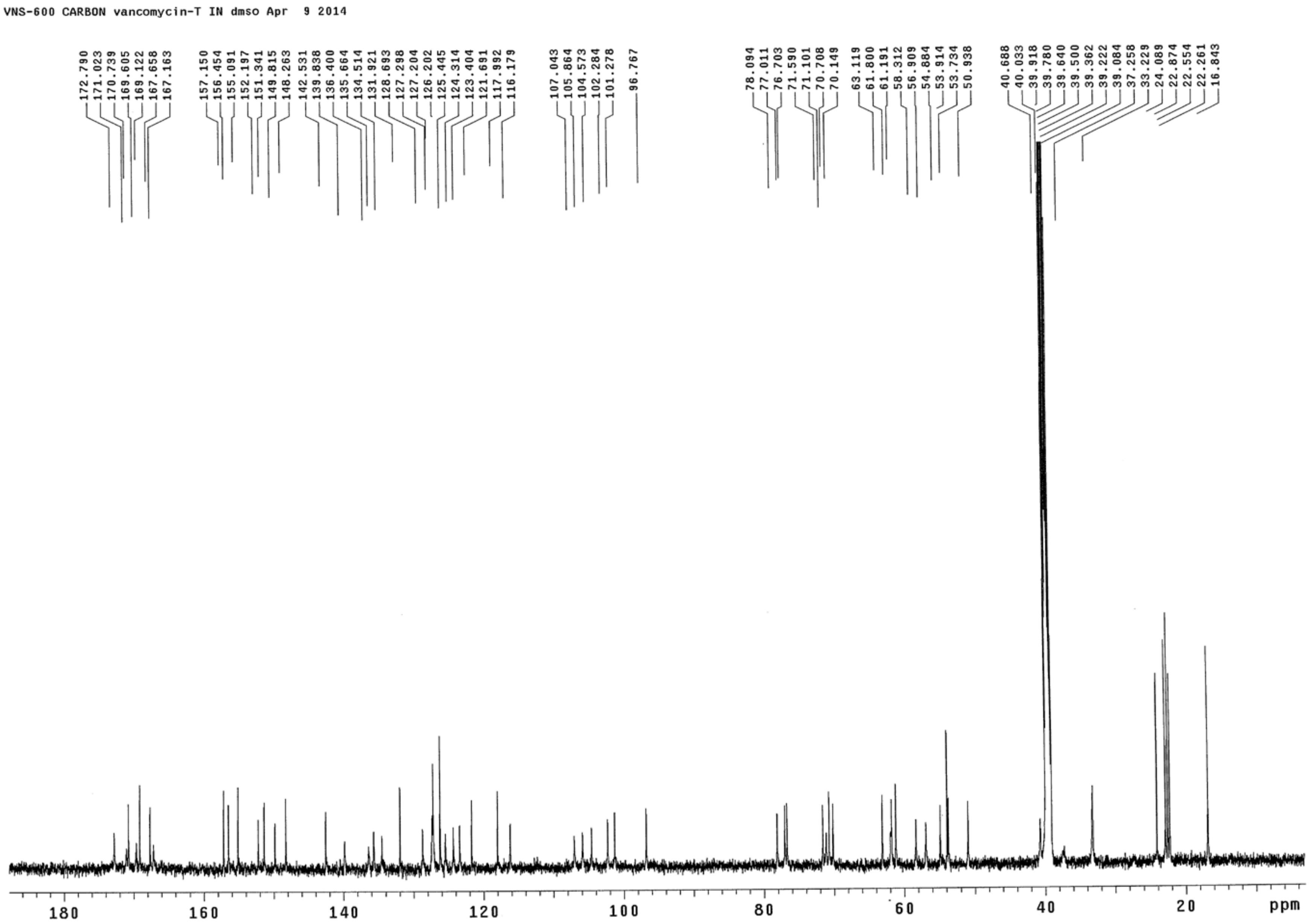

Figure S2. The ^{13}C NMR Spectrum of vancomycin hydrochloride in DMSO-d_6 (600 MHz).

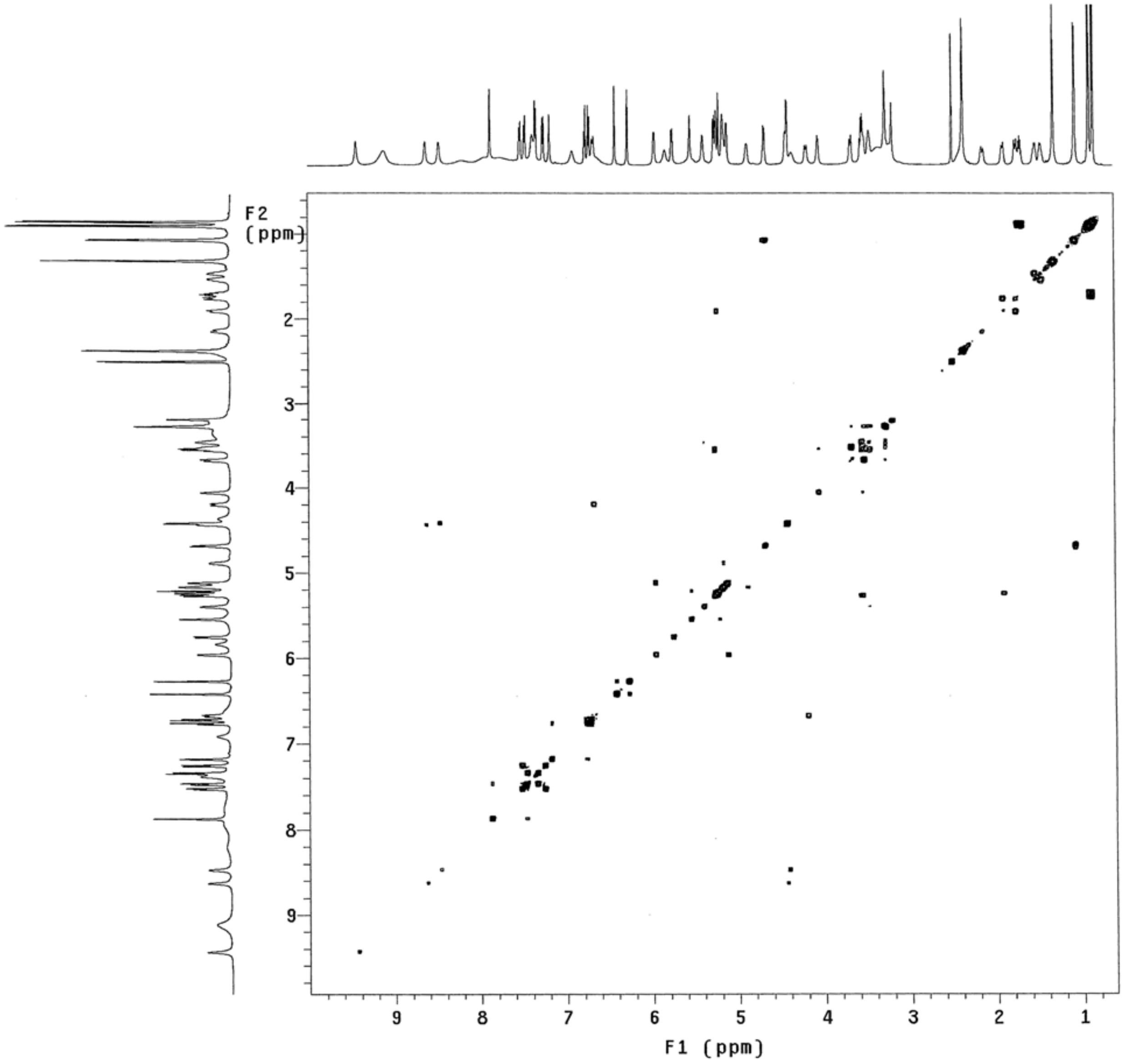

Figure S2. The ^{1}H-^{1}H gCOSY Spectrum of vancomycin hydrochloride in DMSO-d_6 (600 MHz).

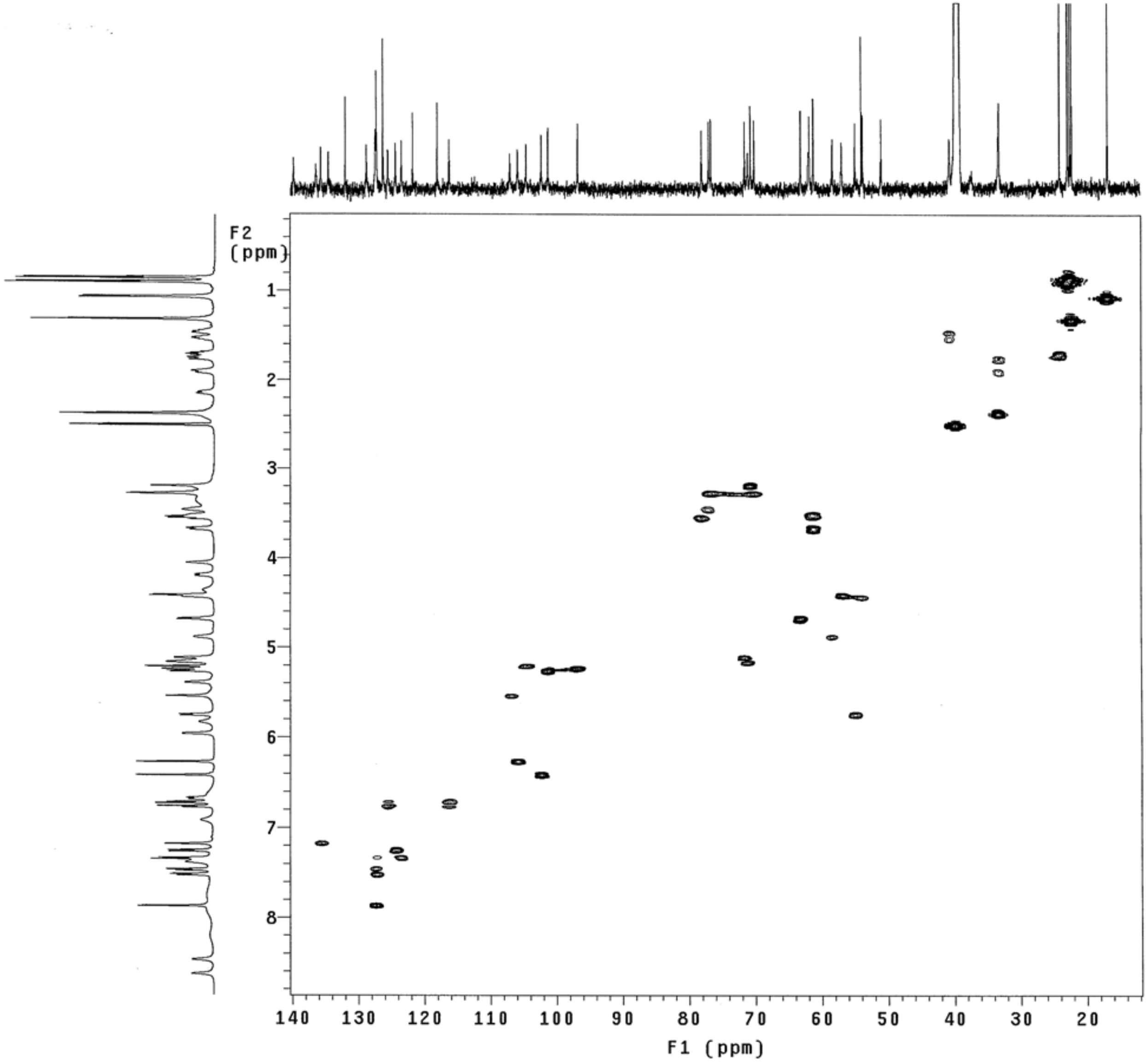

Figure S4. The gHSQC Spectrum of vancomycin hydrochloride in DMSO-d_6 (600 MHz).

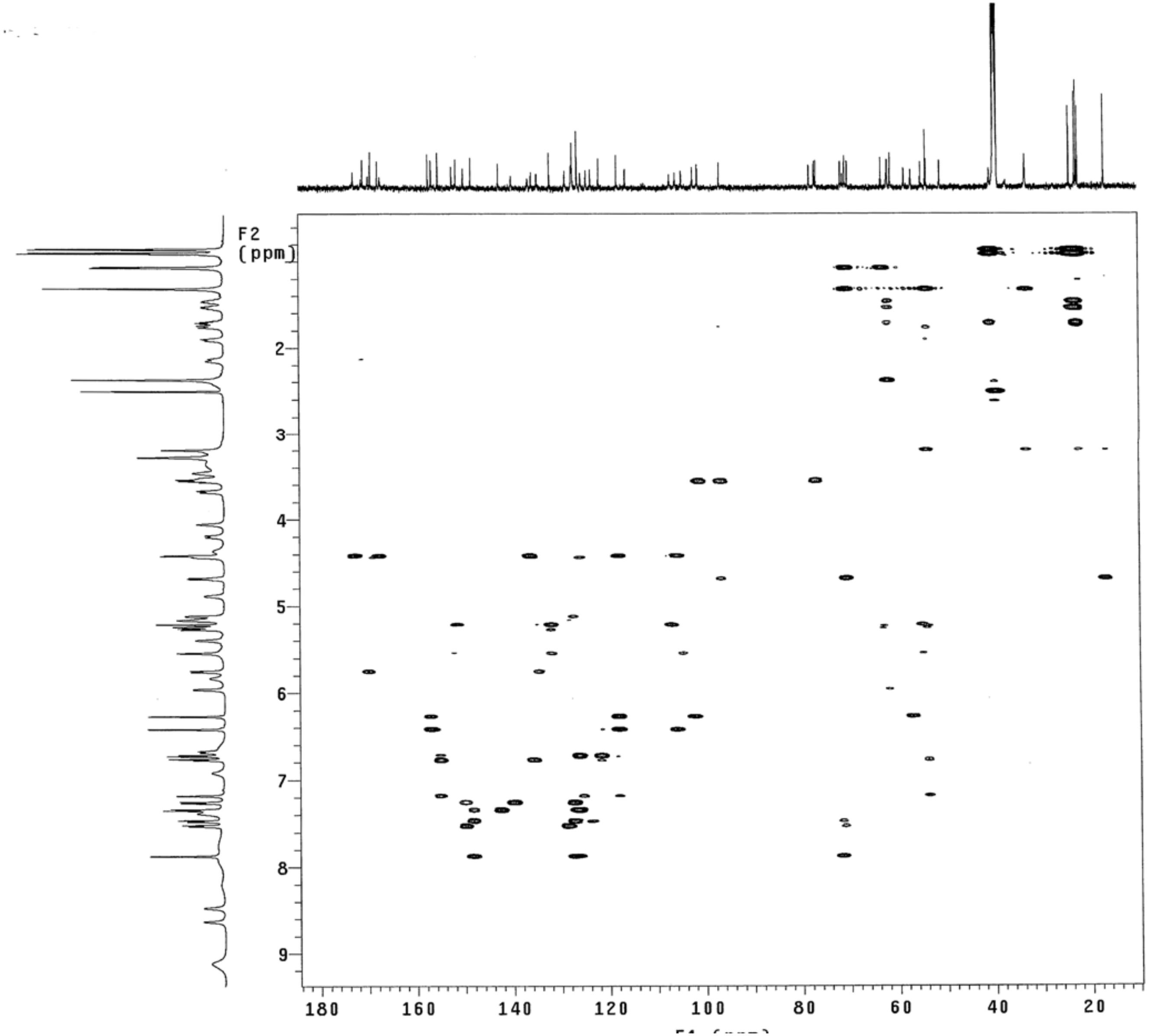

Figure S5. The gHMBC Spectrum of vancomycin hydrochloride in DMSO-d_6 (600 MHz)
